# Development of an Online Reproductive Health Intervention for Individuals with Sickle Cell Disease or Trait

**DOI:** 10.1089/whr.2020.0098

**Published:** 2021-05-21

**Authors:** Versie Johnson-Mallard, Anne Oguntoye, Nyema Eades, Dalal Aldossary, Grace Kuenzli, Miriam O. Ezenwa, Agatha M. Gallo, Diana J. Wilkie

**Affiliations:** ^1^ College of Nursing, University of Florida, Gainesville, Florida, USA.; ^2^ College of Nursing, University of Illinois at Chicago, Chicago, Illinois, USA.

**Keywords:** child health, decision making, infant health, intervention

## Abstract

The purpose of this article is to describe the method of developing an internet-based reproductive options intervention to increase informed reproductive decision-making among individuals with sickle cell disease (SCD) or sickle cell trait (SCT). An interprofessional team of graphics and media specialist, nurses, physicians, and researchers collaborated to develop the intervention. Individuals from the community served as advisory board members who reviewed and advised on webpage design, content, delivery, and media. The intervention was theory based, delivered online, and experientially oriented for young adults of reproductive age with SCD or SCT. The intervention was culturally specific, supporting individuals with SCD or SCT in making informed reproductive decisions about transmission of SCD or SCT to their offspring. The intervention could be strengthened to include content on implementing behaviors concordant with informed reproductive decisions. Health care providers can use the result of this study to enhance their knowledge about the complexity of parenting options.

## Introduction

In the United States, 1 of every 500 African Americans has sickle cell disease (SCD) and 1 of every 12 African Americans has sickle cell trait (SCT).^1^ Between 2000 and 2006, 15,277 American infants were diagnosed with SCD in 44 states.^1^ To treat approximately 100,000 individuals with SCD in the United States, the annual cost is 2.4 billion dollars.^2^ Due to global migration, contributions to the U.S. SCD population include genetic sickle cell pools from Africa, the Middle East, Mediterranean, Central and South America, and India and Hispanic populations.^1^ Individuals with SCD or SCT are at risk for having children with SCD if their reproductive partners have SCT or SCD. Current genetic counseling offered to parents after their child has been born with SCD is reactive. Reactive counseling can be distressing for parents who could have been proactive if armed with current evidence-based reproductive choices when aware of their own sickle cell status. Reproductive health decision interventions are necessary for young adults with SCD or SCT. Unfortunately, there are not many opportunities for young adults with SCD or SCT to receive comprehensive information about sickle cell inheritance and their options for reproduction.

An internet-based reproductive options intervention was designed to facilitate informed reproductive health decisions and behavior among young adults at risk for their children inheriting SCD or SCT.^3^ A randomized controlled trial of the intervention showed statistically significant sustained intervention effects on the knowledge outcome.^3^ However, the intervention effects were not significant for the intention and behavior outcomes,^3^ which suggests the need for a critical appraisal of the intervention. The purpose of this article is to describe the method of developing this reproductive options intervention, including the need for the intervention and its theoretical basis, development process, content, and delivery process. We conclude with several recommendations for future directions.

## Background

Among studies conducted on SCD, very few have focused on reproductive behaviors of young adults living with SCD or SCT.^4^ We identified one study that investigated the efficacy of a clinically delivered, genetic education program for adolescents with SCD.^5^ Porter et al. used a genetic counseling framework with a candy model strategy to convey genetic risks and found that adolescents were able to grasp the basic genetic inheritance concepts of SCD. Other quantitative studies explored the attitudes and beliefs and desire for reproductive health knowledge on SCD or SCT.^5–8^ Each of these studies was conducted in the United States, except for Melaibari et al.'s study, which investigated views of young adults in Saudi Arabia on the national premarital screening program of the country.^9^ Findings were similar across the board, with studies indicating a general lack of knowledge about reproductive options and sickle cell inheritance, and confusion about adequacy of resources for this information. The qualitative studies we identified follow a similar trend—exploring attitudes, beliefs, and reproductive decision-making of individuals with SCD or SCT.^4,10,11^ This body of literature indicates a dearth of interventions focused on the reproductive health information needs of SCD or SCT populations. A gap remains among studies providing reproductive options. An internet-based intervention^3,12,13^ is an exception. Gallo et al.^3^ developed an intervention to educate reproductive-age adults with SCD or SCT on reproductive knowledge (genetic inheritance) of sickle cell. The preconception education intervention included options, such as adoption, preimplantation genetic testing, or not to parent, which could enable individuals to specify a parenting plan in advance of pregnancy. The internet-based reproductive options intervention was based on two guiding theories, the theory of reasoned action (TRA) and Kolb's experiential learning theory (ELT).

### Theoretical basis

**FIG. 1.**
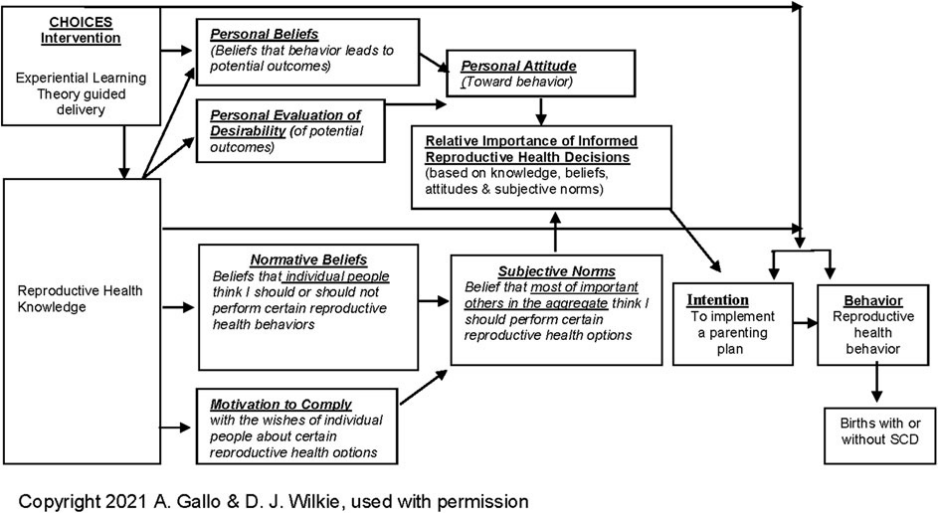
Theory of reasoned action: content bridging. SCD, sickle cell disease.

The TRA is a behavioral change theory shown to aid individuals in changing unhealthy or adopting healthy behaviors.^14^ TRA served as a conceptual basis for the content of the CHOICES intervention. The constructs within TRA (*e.g.*, behavioral beliefs, evaluation of behavioral outcomes, normative beliefs, and motivation to comply) steered the content for the intervention and measures of its effects (Fig. 1). Since the delivery of an intervention is just as important as its content, Kolb's ELT guided the delivery of the intervention as an engaging, interactive, and user-friendly electronic format. Table 1 provides an overview of knowledge and behavior content and is summarized within the four components of the ELT. The TRA and ELT provided a strong conceptual basis for the intervention development process.

**Table 1.** Overview of Knowledge and Behavior Elements of the Intervention: Organized by Kolb's Experiential Learning Theory

Kolb's experiential learning theory	Knowledge and behavior elements
*Concrete experience*	To involve subjects in the experience and begin to ready themselves for behavior change, Dwayne's Story shows two young men walking home from playing on the basketball court after Dwayne is notified that his baby has SCD. They begin talking about it. Dwayne and his partner did not know that they had SCT until they were tested after the baby was diagnosed with SCD. As they talked, Sean realizes that he does not know if he or his partner has the SCT. Sean is concerned about the possibility of having a baby with SCD. Addresses expectations, video model-negative outcomes, and importance of health behavior (SCT testing).
*Focused on the importance of getting tested for SCT*	
*Reflective observation*	After viewing and reflecting on the video, we ask subjects to type in their responses (Your Thoughts) to three related questions about their ideas about SCD: 1. Are you worried about having a baby with sickle cell disease? Why or why not? 2. Have you or your partner been tested for SCT? Why or why not? 3. When do you think a couple with sickle cell disease or trait should talk about having babies? Why talk? Addresses personal risk and potential behaviors for risk reduction.
*Moving from Dwayne and Sean to own personal perspective*	
*Abstract conceptualization*	The Information For You component gives subjects information about becoming a parent, the genetic inheritance of SCD and SCT, and reproductive options (*e.g.*, talking to one's partner about sickle cell status, having a baby naturally, partnering with a person with normal hemoglobin, adopting or fostering children, and medical options such as options to avoid pregnancy, prenatal testing, and artificial reproductive technologies).
*Interactive information about SCD and SCT and developing a parenting plan*	
	To engage subjects in the content and help overcome knowledge gaps about the inheritance of SCD or SCT, available reproductive options, and behavioral change steps needed to implement a parenting plan, the information is displayed in a variety of ways—by text, video clips, audio clips, graphics, cartoons, photos, animations, and testing one's knowledge to reinforce application of what has been learned.
*Active experimentation*	In the People's Experiences component, subjects view many videos where couples talk over their decisions made based on their sickle cell status. These videos generalize content within the context of other couples' decisions and behaviors. For example, couples discuss and model their behavior steps to have their babies naturally or consider other options discussed in the *Abstract conceptualization* component. Subjects choose the video that best characterized their situation and decision. At the end of the program, the computer program generates a parenting plan that recaps the subject's responses to intervention and SCKnowIQ items. The subject indicates if the parenting plan is accurate/inaccurate and receives an electronic copy of the personal parenting plan that is tailored to his/her situation with behaviors listed—those needed for the parenting plan.
*Role models perform behaviors needed to implement various parenting plans. Helps the user take a stand and get a list of steps*	

SCD, sickle cell disease; SCKnowIQ, Sickle Cell Knowledge Intention Questionnaire; SCT, sickle cell trait.

## Intervention Development Process

A team of nurses, researchers, physicians, community members with SCD or SCT, a graphics and media specialist, and a software engineer collaborated in an iterative process to develop the reproductive options intervention. In collaboration with the community members, nurse scientists led the team, developed the content outline, and drafted intervention content and media scripts. Using expertise in genetics of SCD and human reproductive behavior, the research team used the TRA to focus the intervention's content around information an individual with SCD or SCT would need to make informed decisions about a preferred parenting plan. For example, individuals were provided information on the genetic inheritance of sickle cell, birth control options, and general information on adoption or fostering. Extreme care was taken to present all options equally and avoid presenting any option as more desirable. Permanent contraception, adoption, *in vitro* testing, and advanced reproductive techniques were all presented in a nonbiased informative manner. The content emphasized the notion that the individuals and their reproductive partners made the decision about their preferred parenting plan.

After content experts drafted the intervention content, other professionals and individuals with SCD or SCT expertise reviewed the intervention for accuracy and understandability. This review included a general review with mapping of the content to the TRA domains. Intervention content revisions were made based on an iterative series of reviews. Then, the nurse experts guided the graphic and media specialist to illustrate the content and develop the interactive components. The nurse experts also worked with the software engineer to develop the database and create webpages. The media specialist recruited the actors for interactive audio and video components and produced and edited the media, all of which required a considerable amount of strategic planning to ready the materials for the software engineer.

An advisory board of four (4) young adults with SCD or SCT reviewed the media/videos and webpage design. The advisory board reviewed the reproductive options intervention and their suggested changes were integrated and reviewed again to ensure that the revision sufficiently captured all suggestions. The research team tested the prototype intervention with a group of 10 young adults with SCD or SCT using a cognitive interview process, with findings supporting the validity of the intervention.^15^

### Description of the content

The knowledge and behavior content of the internet-based reproductive intervention is theory based, experientially oriented at the primary prevention level, and developed to improve public health, inform reproductive options, and assist with future pregnancy planning. The content is designed specifically for young adults of reproductive age with SCD or SCT to consider their reproductive health behaviors and options. Personal options are presented with preventive possibilities to avoid having a child with SCD. The content is fundamentally designed to improve public understanding of complex processes of genetics and inherited disease. The content involves translating TRA concepts (*italics*) within the constructs of Kolb's ELT **(bold)**: (1) **concrete experience—***case-based learning focused on the beliefs and attitudes about the importance of testing for SCD or SCT before procreation*; (2) **reflective observation**—*translating the case to themselves based on personal evaluation of desirability*; (3) **abstract conceptualization**—*depicting living with SCD through interactive information important for developing a parenting plan based on the relative importance of informed reproductive health decisions and community norms*; and (4) **active experimentation**—*depiction of couples performing behaviors needed to implement their intended parenting plans* (Table 1).

Content was developed to increase the understanding of reproductive risks for genetic transmission of SCD and SCT. The intervention informs of reproductive options, risks, and benefits for the woman with SCD. Individuals are provided up-to-date evidence and a list of possible steps designed to help them make reproductive health decisions (Table 2). The content also informs those who may or may not know their partner's SCT status about the importance of testing as part of understanding the genetic transmission of the disease. The intervention's evidence-based content is intended to change knowledge so that intentions and behaviors are congruent with their parenting plan. The **57** webpages present **14** video clips of couples sharing their choices and modeling their reproductive behaviors (Table 3) as well as **17** graphical animations that display, for instance, SCD genetic inheritance risks and many reproductive options.

**Table 2.** Examples of Behavioral Topics and Content Focused Within the Abstract Conceptualization and Active Experimentation Sections

Behavioral topic	No. of webpages (interactive elements)+no. of videos	Content focus
*Becoming a parent*	1	Guide through the parental options, for example, biological children
Life with SCD or SCT		
*Living with SCD and trait*	1 (3) + 2 videos	Basic information on sickle cell disease complications and precautions for those with SCD/SCT
*Transmitting SCD and trait*	3	Sickle cell inheritance using Punnett squares
*Testing for SCD and trait*	2	Screening measures for SCD/SCT
*Talking about SCD and trait*	1	Making SC status a priority discussion in relationships
*Pregnancy*	2	Possible complications and precautions
Parenting plans		
*Decision-making options*	2	Basic reproductive options
	1	Advanced reproductive options
Experiential learning: actors, videos, and scenarios		
*Actors: Marcus and Alisa*	Video clip	Marcus and Alisa discuss sickle cell status, inheritance, and their decision to adopt.
*Actors: Gwen and Jacquie*	Video clip	Gwen's decision to have a child with SCD
Reproductive options to achieve the parenting plan		
*Pregnancy prevention*	3 (chart)	Family planning methods
*Genetic testing*	1 + 1 video	Prenatal testing information and testimonial of invasive prenatal testing (amniocentesis).
*Abortion*	3	Methods of safe abortion
*ART*	6 + 1 video	Information on ART
*Adoption*	1 (link to adoption resources)	Considerations and resources for adoption
*Foster care*	1 (link to foster care resources)	Considerations and resources for fostering
Acted testimonials from couples		
*Talking about your wishes*	2 + 2 videos	Timing a discussion about SCD/SCT status with a partner
*Both have SCD*	1 + 1 video	Couple with SCD give a testimonial on their decision to start a family
*One has SCD and the other has SCT*	1 + 1 video	Couple discuss their decision to start a family
*Both have SCT*	1 + 1 video	Couple with SCT give a testimonial on their decision to start a family

ART, advanced reproductive techniques.

**Table 3.** Examples of Videos Within the Experiential Learning Activities

*One has SCT and the other has normal hemoglobin*	In this video, the couple tells their story of coming to the decision to have their own biological children. The man revealed his SCT status early in the relationship. The couple spoke with their clinician and discussed their options for having a baby. They decided on biological children since none of the pregnancies would be at risk for a child with SCD.
*Both have SCT: couple breaks up*	This video shows a couple 6 months into their relationship. They both discover that they have SCT. Although they are aware of several options available to them to have children who would not have SCD/SCT, they decide to break up because she would like to have her own biological children and cannot risk the chance of them having SCD, but is wary about the cost of ART.
*Both have SCT: couple decides to stay together*	A short time before their wedding, a couple learns that they both have SCT. Since the woman is currently on birth control and they are both in love with each other, they decide to stay together and figure out their options for having children later. Following their wedding, they meet with a clinician who explains SCD inheritance and available options to them. They decide to think further on this new information and make a decision that will be best for them.
*Both have SCD*	This couple started their relationship knowing that they both had SCD. The woman wanted her own biological children despite knowledge of possible complications. They decided that they did not want their children to have SCD. After speaking with their clinician, the couple decided on *in vitro* fertilization with her egg and the sperm of a man with normal hemoglobin. Although she describes the pregnancy as tough, they are happy with their decision and now have a baby with SCT.

### Delivery process

**FIG. 2.**
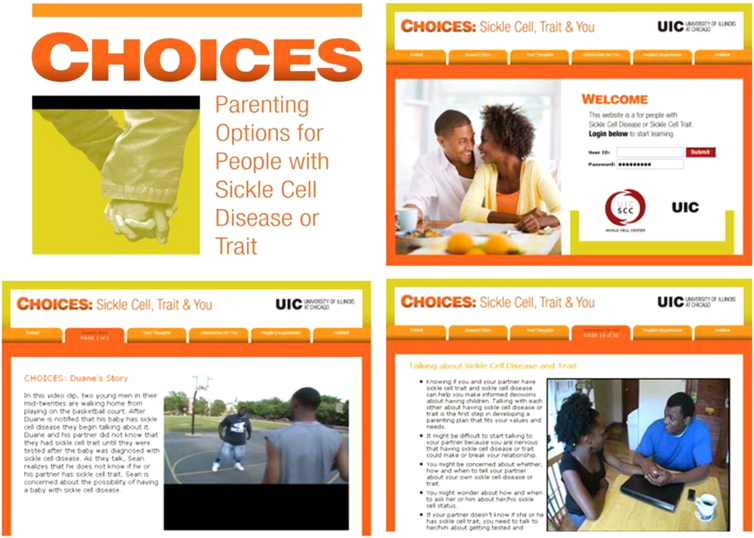
Examples of intervention screens.

The delivery of the intervention begins once the individual signs in and launches the *Pretest*, which needs to be completed before proceeding through the rest of the program (Fig. 2). The pretest is an online, 71-item, Sickle Cell Knowledge Intention Questionnaire (SCKnowIQ) developed to assess reproductive knowledge behaviors and intentions of those with SCD or SCT.^15^ Once the pretest is completed, the participant moves to the introductory video screen that depicts two male actors having a conversation about one man's newborn daughter born with SCD and the problem of neither parent being aware of their SCT status. The inclusion of this video is in line with the concept of *concrete experience* in ELT, which is intended to give the learners a tangible experience as they begin to engage with education materials (Table 1).

The third tab (Fig. 2) on the user's intervention screen is *Your Thoughts*, which leads directly to the *reflective observation*. *Your Thoughts* asks the participants pointed questions related to their ideas and beliefs about SCD to personalize the *concrete experience* within the learner's normative beliefs and behavioral beliefs, concepts within TRA (Fig. 1). This personal reflection allows individuals to contemplate where they are with parental planning and what they may hope to achieve.

The fourth tab, *Information for You*, contains evidence-based information related to becoming a parent, genetic inheritance of SCT and SCD, and possible reproductive options such as having a baby naturally, adopting or fostering, or having prenatal testing. The information is presented in a myriad of ways such as text, audio/video clips, animations, photos, and testing one's knowledge to reinforce information learned (Table 2). This section provides interactive information about SCD and SCT while aiding participants in thinking about a parenting plan. *Information for You* content is modeled after ELT's *abstract conceptualization* component and bridges any gaps in knowledge, which is an important component of behavior congruent with the parenting plan (Fig. 2).

In the fifth tab, *People's Experiences*, participants are presented with five scenarios, which may mirror the situation they find themselves in. The *active experimentation* component of ELT appears in the People's Experiences section and users can choose a scenario that best characterizes their situation and decision. This section provides role models of behaviors to implement various parenting plans and encourages individuals to make a list of steps required to reach their desired goals, an important factor of behaviors congruent with their parenting plan (Table 3).

The last section is the post-test, which like the pretest includes items from the SCKnowIQ. Once these items have been completed, the program generates a parenting plan that reflects the user's responses to the program and SCKnowIQ items. The user indicates if the parenting plan is an adequate representation of their goals. The parenting plan is tailored to each user's situation, listing behaviors in which they should engage to achieve their desired goals.

The experience of the intervention can be tailored to the participant's desired technology settings. For example, settings include a read-aloud function, which presents the information on the screen to the users in either a male or female voice, or the option for on-screen text without audio. These settings can be tailored at any point during the program.

## Recommendations for Future Directions

Strategic use of the internet and mobile devices is ideal for dissemination of health information.^16^ In this article, we describe development and content of a reproductive options intervention focused on improving knowledge, intention, and behaviors to implement a parenting plan in advance of pregnancy. This intervention was designed for delivery to young adults with SCD or SCT through the internet and mobile technology. The preferred method of communication among young adults appears to be the internet and mobile devices,^16^ which positions this intervention to have an impact on a public health concern. Prior studies of the intervention showed that dissemination of tailored age- and population-appropriate information is possible with mobile technologies.^3,13,15^ Furthermore, interventions supported by digital technology also allow for global dissemination.

Critical analysis of the publications on intervention effects provides important insights for refining the intervention. Analysis of the theoretical basis, development process, content, and delivery process suggests that the intervention is an exceptional tailored intervention designed to educate young adults living with SCD/SCT about their reproductive options. The format and content of this educational intervention are optimal for future social media and SMS text formatting. The published evaluative comments from study participants^17,18^ indicate that the content needs to be strengthened in the area of reproductive health concerns of same-sex couples. The nonsignificant improvement in behavior change to implement the individual's parenting plan indicates that the reproductive options intervention could be strengthened to include educational content on implementing behaviors concordant with informed reproductive decisions.

Critical analysis of the publications also provides important insights for designing another randomized controlled trial. The study design to test intervention efficacy requires careful consideration of eligibility criteria. Clearly, there is a need to recruit study participants whose reproductive partners' status puts them at risk for having a child with SCD and who express the intention of soon having a child free of SCD. Inclusion of individuals not at risk or not intending to have a child free of SCD, who do not need to change their behavior to be concordant with their reproductive decisions, decreases the power to detect an intervention effect among individuals who do need behavior change to achieve their own goals.

The reproductive options intervention holds promise to support individuals with SCD and SCT in making reproductive decisions and, with enhancements, could assist individuals or couples in implementing behaviors concordant with their informed reproductive decisions. A future application of the intervention could enhance health care provider knowledge about the complexity of information needed for parenting plan options and translate evidence-based information to the stakeholders.

## Abbreviations Used

ELT: experiential learning theory
SCD: sickle cell disease
SCKnowIQ: Sickle Cell Knowledge Intention Questionnaire
SCT: sickle cell trait
TRA: theory of reasoned action
